# Short-term disengagement from early intervention service for first-episode psychosis: findings from the “Parma Early Psychosis” program

**DOI:** 10.1007/s00127-023-02564-3

**Published:** 2023-10-13

**Authors:** Lorenzo Pelizza, Emanuela Leuci, Emanuela Quattrone, Silvia Azzali, Simona Pupo, Giuseppina Paulillo, Pietro Pellegrini, Marco Menchetti

**Affiliations:** 1 Department of Biomedical and Neuromotor Sciences, “Alma Mater Studiorum”, Università di Bologna, Viale Pepoli, 5, 40126 Bologna, BO Italy; 2 Department of Mental Health and Pathological Addiction, Azienda USL di Parma, Parma, PR Italy; 3 Department of Mental Health and Pathological Addiction, Azienda USL-IRCCS di Reggio Emilia, Reggio Emilia, RE Italy; 4 Pain Therapy Service, Department of Medicine and Surgery, Azienda Ospedaliero-Universitaria di Parma, Parma, PR Italy

**Keywords:** Disengagement, Outcome, Early intervention in psychosis, First-episode psychosis, Drop-out, Follow-up

## Abstract

**Purpose:**

Service disengagement is a major concern for “Early Intervention in Psychosis” (EIP). Indeed, identifying predictors of engagement is crucial to maximize mental healthcare interventions in first-episode psychosis (FEP). No Italian study on this topic has been reported to date. Thus, the aims of this investigation were: (1) to examine short-term disengagement rate in an Italian population of FEP patients treated within an EIP service across a 1-year follow-up period, and (b) to assess the most relevant predictors of disengagement in the first year of treatment.

**Methods:**

All participants were young FEP help-seeking patients, aged 12–35 years, enrolled within the “Parma Early Psychosis” (Pr-EP) protocol. At baseline, they completed the Positive And Negative Syndrome Scale (PANSS), the Health of the Nation Outcome Scale (HoNOS) and the Global Assessment of Functioning (GAF) scale. Univariate and multivariate Cox regression analyses were used.

**Results:**

496 FEP individuals were enrolled in this research. Across the follow-up, a 16.5% prevalence of short-term disengagement was found. Particularly robust predictors of service disengagement were poor baseline treatment non-adherence, living with parents and the presence of brief psychotic disorder or schizophreniform disorder at entry.

**Conclusion:**

About 16% of FEP patients disengaged the Pr-EP program within the first year of treatment. A solution to reduce disengagement and/or to favor re-engagement of these subjects might be to remain on EIP program caseloads allowing the option for low-intensity support and monitoring, also via remote technology.

## Introduction

*Service disengagement* is a clinical challenge that afflicts mental healthcare systems [1]. Indeed, engagement prevalence rates in mental health centers are lower than those reported in other medical services [2]. It has been found that 40–50% of patients with severe mental illness (i.e., schizophrenia spectrum disorders and bipolar disorder) receiving specialist interventions within mental healthcare services disengage from treatment, contributing to poor outcomes and escalating health care costs [3].

*Early intervention in psychosis* (EIP) services providing evidence-based treatments lead to better outcomes in symptom remission, relapse prevention and socio-occupational recovery than does usual psychiatric care [4]. However, the therapeutic benefits of EIP (usually offered for the initial 2–3 years following a first-episode psychosis [FEP]) are widely influenced by the degree to which patients engage in treatment. Indeed, FEP patients who disengage or are only superficially engage are at greater risk of relapse [5]. In this respect, a recent meta-analysis on EIP programs reported a 15.6% pooled prevalence rate of disengagement, with very high heterogeneity across studies (also due to various definitions of engagement and different length of follow-up) [6]. Indeed, two relevant systematic reviews on the strength of engagement showed that disengagement rates within EIP services ranged from 1% to 53% [7], despite ongoing therapeutic need [7, 8].

In addition, adolescents and young adults “per se” are at high risk for disengaging from mental healthcare services, especially during the adolescent–adult transition age [9]. This is of particular concern for young people with FEP, given that its peak onset most often occurs during adolescence and young adulthood [10], and its outcome trajectories are established relatively early (usually during the first 2–5 years from presentation) [11].

Furthermore, targeting and monitoring engagement levels are usually considered as important measures of the *quality of care*, also within EIP services [12]. Currently, there are no universally accepted definitions for mental healthcare engagement [13]. Indeed, it is typically assessed using proxies (such as treatment drop-out or therapeutic alliance), with disengagement definitions ranging from “when patients actively refused any contact with the treatment staff and were not traceable” [14] to “terminated treatment despite therapeutic need” [15]. However, these proxies probably are simplistic approaches to understand a more complex, longitudinally dynamic and multidimensional phenomenon that encompasses multiple factors (e.g., acceptance of a need for help, therapeutic alliance, satisfaction with the therapy already received, mutual working towards shared goals, variations in catchment population served, differences in service models, changes in relation to stages of treatment and patient needs) [16, 17].

The lack of consensus about how to conceptually define disengagement in EIP services has led to large variations in its reported prevalence rates across studies [18] and to inconsistent (sometimes conflicting) results on its putative predictive factors [19]. Therefore, further research (especially with a longitudinal design) is needed to clarify these mixed findings and to increase our knowledge on those predictors that are most important for disengagement from EIP services, especially in the early stages of intervention (i.e., the “short-term service disengagement”) [20, 21]. In this respect, several studies included in meta-analyses on service disengagement [6, 7] were conducted on retrospective cohorts. Among prospective cohorts, only few investigations considered large FEP populations with 500 or more participants [3, 22, 23].

Starting from this background, the *aims* of this investigation were: (a) to examine short-term disengagement rate in a large Italian population of FEP patients treated within an EIP service across a 1-year follow-up period, and (b) to assess the most significant predictors of disengagement in the first 12 months of intervention. To the best of our knowledge, no Italian study on engagement in EIP programs has been reported in the literature to date.

## Methods

### Sample and setting

Participants were FEP adolescents and young adults entered the “Parma Early Psychosis” (Pr-EP) program between January 2013 and December 2020. The *Pr-EP program* is an EIP protocol implemented as a diffuse infrastructure in all adult and adolescent mental healthcare services of the Parma Department of Mental Health, in Northern Italy [24].

For the specific purpose of this research, *inclusion criteria* were: (a) age 12–35 years; (b) specialist help-seeking request; (c) presence of FEP within one of the following DSM-5 diagnoses: schizophrenia, bipolar disorder with psychotic features, major depressive disorder with psychotic features, delusional disorder, brief psychotic disorder, schizophreniform disorder, and psychotic disorder not otherwise specified [25]; (d) enrollment within the Pr-EP program [26]; and (e) a duration of untreated psychosis (DUP) of < 2 years. This specific DUP (defined as the time interval [in months] between the onset of overt psychotic symptoms and the first antipsychotic prescription) [27] was selected because it is the usual limit to provide effective interventions within the EIP paradigm [28].

*Exclusion criteria* were: (a) past DSM-5 affective or non-affective psychotic episode; (b) past exposure to antipsychotic medication; (c) known intellectual disability (i.e., IQ < 70); and (d) neurological disorder or any other medical condition presenting with psychiatric symptoms. Past exposure to antipsychotic drug (i.e., at any dosage and at any time before the Pr-EP enrollment) was considered as “functional equivalent” of a past psychotic episode [29]. Indeed, the EIP paradigm psychometrically defined the psychotic threshold as “essentially that at which antipsychotic medication would probably be started in common clinical practice” [30].

Based on symptom severity, the Pr-EP protocol offered a 2-year comprehensive *treatment package* including a psychopharmacological therapy and a multi-element psychosocial intervention (combining individual psychotherapy inspired on cognitive-behavioral principles, psychoeducational sessions for family members and a recovery-oriented case management), in accordance with the current EIP guidelines [31, 32]. Low-dose atypical antipsychotic drugs were used as first-line treatment [33]. Selective serotonin reuptake inhibitors and benzodiazepines could also be used in case of depression, anxiety or insomnia [34].

Individual psychotherapy was developed on the cognitive-behavioral model of Garety and colleagues [35] for psychosis. Family intervention was based on the cognitive-behavioral model by Falloon and co-workers [36] for psychotic disorders. As for case management, each individual/family received a dedicated case manager coordinating all the planned interventions, including those aimed at an early, recovery-oriented rehabilitation and at promoting job and social inclusion [29, 37].

### Instruments

For the specific goals of this study, a *sociodemographic/clinical chart* (collecting information on gender, age at entry, ethnic group, migrant status, years of education, occupation, living status, past specialist contact, previous hospitalization, current substance abuse, DUP, previous suicide attempt, and acceptance of psychopharmacological and/or psychosocial treatments) was retrospectively completed at baseline by Pr-EP team members (i.e., psychiatrists or clinical psychologists) based on medical record [38]. Specifically, we defined “suicide attempt” as a potentially injurious, self-inflicted behavior without a fatal outcome for which there was (explicit or implicit) evidence of intent to die [39].

The *DSM-5 diagnosis* was formulated at entry by at least two trained Pr-EP team members using the Structured Clinical Interview for DSM-5 mental disorders (SCID-5) [40]. The presence of *FEP* was also confirmed using the psychometric criteria of the Comprehensive Assessment of At-Risk Mental States (CAARMS), authorized Italian version [30, 41].

The psychopathological and functioning assessment included the Positive And Negative Syndrome Scale [42], the Global Assessment of Functioning (GAF) scale [25] and the Health of the Nation Outcome Scale (HoNOS) [43]. These instruments were completed by trained Pr-EP team members at entry. Regular scoring workshops and supervision sessions were used to ensure the inter-rater reliability of these scales [44].

The *PANSS* is a semi-structured clinical interview specifically developed to assess psychosis psychopathology, also in FEP patients [45]. As proposed by Shafer and Dazzi [46], we considered 5 main psychopathological factors: “Negative Symptoms”, “Affect” (“Depression/Anxiety”), “Positive Symptoms” “Disorganization” and “Resistance/Excitement-Activity”. We used the Italian version of the PANSS [47] that showed good psychometric properties also in young Italian FEP populations [48].

The *GAF* is a widely used scale for the assessment of daily functioning in individuals with severe mental illness. The Italian version of the GAF was commonly used also in young Italian people with FEP [49].

The *HoNOS* was specifically developed to evaluate social and clinical outcomes in people with severe mental illness. As proposed by Golay and co-workers [50], we considered 4 main outcome domains: “Psychiatric Symptoms”, “Impairment”, “Social Problems” and “Behavioral Problems”. We used the Italian version of the HoNOS [51] that showed good psychometric properties also in young Italian individuals with FEP [52, 53]. As indicated in the Mental Health Clustering Tool (*MHCT*) [54], together with the HoNOS items, we also considered 5 historical ratings on events that could remain relevant to the current plan of care. These historical scores include an “Engagement” item that specifically rates the patients’ motivation and understanding of their problems, the acceptance of their care/treatment and the ability to relate to care staff [55]. In other words, it considers the subjects’ ability, willingness or motivation to engage in their care/treatment appropriately, to agree personal goals and to attend appointments [56].

### Disengagement groups

As suggested by Robson and Greenwood in the only meta-analysis currently published on the strength of engagement in FEP patients [6], we defined service disengagement as “complete lack of contact or untraceable for at least 3 months despite a need of treatment, counted from the date of the last face-to-face meeting with the clinical staff”. This definition included FEP people who actively refused further contact with the treatment staff and were no longer traceable [14], those who did not return phone calls or not attend appointments for at least 3 months [57] and those who prematurely exit EIP treatments against clinicians’ advice [58]. FEP patients who moved out of our catchment area or are appropriately discharged (i.e., clinically improved and transferred to other, private or public, generalist mental healthcare professionals) were excluded from analysis [6]. Those who died or were imprisoned were also excluded from analyses on the basis that any conclusions about engagement could not be drawn from these events [6]. FEP participants meeting criteria for our definition of service disengagement were included in the FEP/SD + subgroup. The remaining individuals were grouped in the FEP/SD- subsample.

For short-term service disengagement condition in the FEP total group, we finally examined any significant association with sociodemographic, functioning, psychopathological and clinical features at entry, as well as with baseline acceptance of Pr-EP treatment proposals.

### Statistical analysis

Data were analyzed using the Statistical Package for Social Science (SPSS) for Windows, version 15.0 [59]. Statistical analyses were two-tailed, with a significance level set at 0.05.

Cumulative proportional risk rates of short-term service disengagement condition were calculated using the Kaplan–Meier survival analysis, which is able to take into account the time of survival (in months) among the patients entered the 1-year follow-up period [60]. Significant associations of short-term service disengagement with acceptance of Pr-EP therapeutic proposals, sociodemographic and clinical characteristics at baseline were explored in the FEP total sample using Cox regression analysis. Specifically, after having previously checked that the proportionality-of-hazards assumption was met [61], univariate models were fitted for each potential predictive variable. The predictors resulted statistically significant were then put as covariates into a multivariate Cox regression analysis to test the strongest predictive parameters for short-term service disengagement. This 2-step method allowed us to adapt the number of covariates to the size of our FEP population, keeping a ratio equal to at least 1:20 (i.e., 20 participants for each covariate) [62].

## Results

**Fig. 1:**
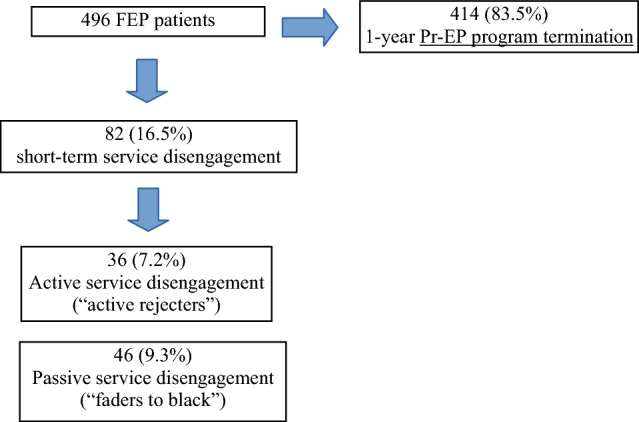
Prevalence rates of short-term service disengagement across a 1-year follow-up period in the FEP total sample (*n* = 496). FEP = first-episode psychosis, Pr-EP = “Parma Early Psychosis” program; “active rejecters” = FEP participants who actively refused contact with the treatment staff and were not traceable for at least 3 months; “faders to black” = FEP participants who simply did not return phone calls or not attend appointments for at least 3 months, despite ongoing therapeutic need (i.e., they did not explicitly refused treatment, but silently dropped out the Pr-EP protocol without being no longer traceable). Cumulative prevalence rates are reported

A total of 496 FEP patients were enrolled in this research. Eighty-two (16.5%) of them showed a short-term service disengagement and were included in the FEP/SD+ subgroup (Fig. 1). The remaining 414 participants concluded the 1-year follow-up period and were included in the FEP/SD-subsample. Among “early disengagers”, 36 individuals actively refused contact with the treatment staff and were no longer traceable against clinician’s advice (“active rejecters”) and 46 simply did not return phone calls or not attend appointments for at least 3 months despite ongoing therapeutic need (“faders to black—i.e., they did not explicitly refused treatment, but silently dropped out the Pr-EP protocol without being no longer traceable). Kaplan–Meier survival analysis results confirmed a 1-year estimated cumulative short-term service disengagement rate of 0.165 (Table 1).

**Table 1:** Kaplan–Meier analysis results on short-term service disengagement across a 1-year follow-up period in the FEP total sample (*n* = 496)

Variable	1-Cumulative proportion surviving at the time	
	Estimate	SE
Short-term service disengagement rate	0.165	0.017
Active short-term service disengagement rate (“active rejecters”)	0.075	0.012
Passive short-term service disengagement rate (“faders to black”)	0.096	0.013

FEP = first-episode psychosis; “active rejecters” = FEP participants who actively refused contact with the treatment staff and were not traceable for at least 3 months; “faders to black” = FEP participants who did not return phone calls or not attend appointments for at least 3 months, despite ongoing therapeutic need (i.e., they did not explicitly refused treatment, but silently dropped out the Pr-EP protocol without being no longer traceable). Estimate and standard error (SE) values are reported

In the FEP total sample, DSM-5 diagnoses at entry were schizophrenia (*n* = 256; 51.6%), affective psychosis (*n* = 142; 28.6%), brief psychotic disorder (*n* = 70; 14.1%), psychotic disorder not otherwise specified (*n* = 21; 4.2%) and schizophreniform disorder (*n* = 7; 1.4%).

In the FEP total group, short-term service disengagement was significantly predicted by living with parents, migrant status, previous suicide attempt, low baseline prescription of antipsychotic and/or antidepressant medication, low baseline acceptance of psychosocial interventions (i.e., individual psychotherapy, family psychoeducation and case management), diagnosis of brief psychotic disorder or schizophreniform disorder at entry, and high baseline HoNOS “Engagement (historical)” item score (Table 2). Protective factors for short-term service disengagement in our FEP population were marriage/living with partners and diagnosis of schizophrenia at entry. Statistical trends for prediction of short-term service disengagement (i.e., 0.05 < *p* < 0.01) were also found for female gender, low baseline PANSS “Negative Symptoms”, “Disorganization” and “Resistance/Excitement-Activity” factor scores, and high baseline GAF score.

**Table 2:** Univariate Cox proportional-hazard models for short-term (1-year) service disengagement in the FEP total sample (*n* = 496)

Variable	FEP/SD+ (*n* = 82)	FEP/SD− (*n* = 414)	Statistic test		
			HR	95% CI	*p* value
Gender (female)	38 (46.3%)	146 (35.3%)	1.513	0.980–2.336	0.061
Ethnic group (white Caucasians)	64 (78%)	349 (84.3%)	1.482	0.878–2.500	0.14
Migrant status	20 (24.4%)	60 (14.4%)	0.543	0.328–0.899	**0.018**
Age (at entry)	24.67 ± 6.28	25.55 ± 6.20	0.982	0.948–1.018	0.324
Education (in years)	11.13 ± 2.59	11.56 ± 2.90	0.953	0.884–1.028	0.211
*Civil status*					
Single	57 (69.5%)	254 (61.4%)	0.801	0.111–5.786	0.826
Married/partnership	24 (29.3%)	157 (37.9%)	0.686	0.426–1.105	0.121
Separated/divorced	1 (1.2%)	3 (0.7%)	1.248	0.173–9.013	0.826
*Living status*					
Alone	7 (8.5%)	27 (6.5%)	0.722	0.333–1.566	0.409
Living with partners	40 (48.8%)	354 (85.5)	5.125	3.319–7.916	**0.0001**
Living with parents	35 (42.7%)	33 (8.0%)	0.199	0.127–0.313	**0.0001**
*Occupation*					
Unemployed	42 (51.2%)	218 (52.7%)	0.9	0.529–1.533	0.83
Employed	20 (24.4%)	93 (22.5%)	1.111	0.652–1.892	0.699
Student	20 (24.4%)	103 (24.9%)	0.996	0.585–1.697	0.989
DUP (in weeks)	9.30 ± 10.53	9.96 ± 9.86	0.993	0.970–1.016	0.535
Previous hospitalization	34 (41.5%)	183 (44.2%)	1.118	0.721–1.735	0.618
Previous mental health compulsory treatment	10 (12.2%)	41 (9.9%)	0.816	0.421–1.581	0.547
Previous specialist contact	28 (34.1%)	177 (42.8%)	1.415	0.897–2.234	0.136
Previous suicide attempt	9 (11%)	17 (4.1%)	0.373	0.187–0.754	**0.005**
Substance abuse at entry	31 (37.8%)	162 (39.1%)	1.078	0.690–1.684	0.742
Baseline AP prescription	63 (76.8%)	360 (87.0%)	1.929	1.155–3.223	**0.012**
Baseline equivalent dose of risperidone (mg/day)	2.94 ± 2.23	3.60 ± 2.64	0.881	0.769–1.008	0.066
Baseline LAI AP prescription	10 (12.2%)	36 (8.7%)	1.37	0.707–2.655	0.351
Baseline AD prescription	8 (9.8%)	82 (19.8%)	2.139	1.032–4.437	**0.041**
Baseline MS prescription	11 (13.4%)	57 (13.8%)	1.047	0.555–1.975	0.888
Baseline BDZ prescription	26 (31.7%)	146 (35.3%)	1.194	0.750–1.902	0.454
Baseline acceptance of individual psychotherapy	16 (19.5%)	359 (86.7%)	18.041	10.427–31.216	**0.0001**
Baseline acceptance of family psychoeducation	15 (18.3%)	281 (67.9%)	7.949	4.539–13.922	**0.0001**
Baseline acceptance of case management	20 (24.4%)	350 (84.5%)	12.588	7.589–20.897	**0.0001**
*Baseline DSM-5 diagnosis*					
Schizophrenia	34 (41.5%)	222 (53.6%)	0.62	0.400–0.962	**0.033**
Affective psychosis	20 (24.4%)	122 (29.5%)	1.294	0.782–2.142	0.316
Brief psychotic disorder	18 (22.0%)	52 (12.6%)	0.519	0.308–0.876	**0.014**
Psychotic disorder NOS	3 (3.7%)	18 (4.3%)	1.173	0.370–3.716	0.786
Schizophreniform disorder	7 (8.5%)	0 (0.0%)	49.27	21.209–114.475	**0.0001**
*PANSS*					
Positive symptoms	18.90 ± 6.60	17.55 ± 6.06	1.034	0.983–1.086	0.194
Negative symptoms	21.73 ± 9.97	24.68 ± 8.54	0.966	0.932–1.002	0.062
Disorganization	18.88 ± 7.46	21.31 ± 7.56	0.959	0.916–1.003	0.066
affect	16.41 ± 6.00	16.09 ± 5.41	1.011	0.956–1.069	0.701
Resistance/excitement-activity	8.49 ± 3.42	10.11 ± 4.96	0.927	0.860–1.000	0.051
PANSS total score	87.88 ± 23.69	93.02 ± 23.41	0.992	0.978–1.005	0.22
G8 uncooperativeness	2.39 ± 1.50	2.54 ± 1.76	0.948	0.787–1.142	0.572
G12 lack of judgment/insight	3.46 ± 1.95	3.25 ± 1.95	1.049	0.899–1.223	0.545
GAF	47.13 ± 12.04	44.14 ± 10.04	1.025	0.996–1.054	0.094
*HoNOS*					
Behavioral problems	3.73 ± 2.71	3.84 ± 2.42	0.983	0.899–1.074	0.7
Impairment	3.05 ± 2.36	3.22 ± 2.07	0.968	0.872–1.075	0.547
Psychiatric symptoms	10.71 ± 3.42	10.07 ± 3.30	1.052	0.983–1.126	0.143
Social problems	7.37 ± 4.04	7.75 ± 3.84	0.977	0.923–1.034	0.42
Total score	24.85 ± 9.67	24.88 ± 8.38	0.999	0.974–1.025	0.955
“Engagement (historical)” item subscore	1.55 ± 1.27	1.09 ± 1.28	1.264	1.083–1.474	**0.003**

FEP, first-episode psychosis; SD, short-term service Disengagement; FEP/SD +, FEP patients with SD; FEP/SD-, FEP patients without SD; DUP, duration of untreated psychosis; AP, antipsychotic medication; LAI AP, long-acting injection antipsychotic medication; AD, antidepressant medication; MS, mood Stabilizer; BDZ, benzodiazepine; DSM-5, Diagnostic and Statistical Manual of mental disorders—5th Edition; PANSS, Positive And Negative Syndrome Scale; GAF, Global Assessment of Functioning; HoNOS, Health of the Nation Outcome Scale; HR, Hazard Ratio, 95% CI, 95% confidence intervals for HR; p, statistical significance. Frequencies (and percentages) and mean ± standard deviation are reported. Statistically significant *p* values are in bold

Multivariate Cox regression analysis results showed that living with parents, low baseline acceptance of psychosocial interventions and diagnosis of brief psychotic disorder or schizophreniform disorder at entry were the statistically strongest predictive factors of short-term service disengagement in the FEP total sample (Table 3). The first two robust predictive factors were also confirmed in the multivariate Cox analysis including those variables having previous univariate statistical trends (Table 4).

**Table 3:** Multivariate Cox proportional-hazards models for short-term (1-year) service disengagement in the FEP total sample (*n* = 496)

Variable	*B*	SE	Wald	*df*	*p*	HR	95% CI for HR	
							Lower	Upper
Migrant status	0.445	0.284	2.453	1	0.117	1.56	0.894	2.723
Living with parents	0.611	0.265	5.305	1	**0.021**	1.842	1.095	3.097
Baseline AP prescription	− 0.291	0.272	1.152	1	0.283	0.747	0.439	1.272
Baseline AD prescription	− 0.083	0.392	0.045	1	0.833	0.92	0.427	1.985
Previous suicide attempt	0.588	0.392	2.253	1	0.133	1.8	0.835	3.877
Baseline DSM-5 diagnosis of brief psychosis or schizophreniform disorder	0.736	0.262	7.89	1	**0.005**	2.088	1.249	3.49
Baseline acceptance of psychosocial intervention	− 3.262	0.285	131	1	**0.0001**	0.038	0.022	0.067
Overall (score): χ^2^ = 429.556, *df* = 8, *p* = 0.0001								

FEP, first-episode psychosis; AP, antipsychotic; AD, antidepressant; DSM-5, Diagnostic and Statistical Manual for mental disorders, 5th Edition; Baseline acceptance of psychosocial interventions, baseline acceptance of at least individual psychotherapy, family psychoeducation or case management; *B*, regression coefficient; SE, Standard Error, Wald, Wald statistic value; *df*, degrees of freedom; *p*, statistical significance; HR, Hazard Ratio; 95% CI, 95% Confidence Intervals for HR. Statistically significant *p* values are in bold

**Table 4:** Multivariate Cox proportional-hazard models for short-term (1-year) service disengagement in the FEP total sample (*n* = 496), including variables with a statistical trend (0.05 < *p* < 0.01) in previous univariate Cox proportional-hazard models (see Table 3)

Variable	*B*	SE	Wald	*df*	*p*	HR	95% CI for HR	
							Lower	Upper
Gender (male)	− 0.66	0.37	3.182	1	0.074	0.517	0.25	1.067
Migrant status	− 0.033	0.665	0.002	1	0.96	0.967	0.263	3.562
Living with parents	1.817	0.441	16.979	1	**0.0001**	6.152	2.592	14.599
Previous suicide attempt	− 0.057	1.036	0.003	1	0.956	0.944	0.124	7.196
Baseline AP prescription	− 0.106	0.585	0.033	1	0.857	0.9	0.286	2.83
Baseline AD prescription	− 0.742	0.637	1.355	1	0.244	0.476	0.137	1.661
Baseline DSM-5 diagnosis of brief psychosis or schizophreniform disorder	0.065	0.427	0.023	1	0.88	1.067	0.462	2.465
Baseline acceptance of psychosocial intervention	− 2.742	0.43	40.587	1	**0.0001**	0.064	0.028	0.15
PANSS “Negative symptom” factor	− 0.01	0.024	0.165	1	0.684	0.99	0.946	1.037
PANSS “Disorganization” factor	− 0.023	0.027	0.72	1	0.396	0.977	0.926	1.031
PANSS “Resistance/Excitement/Activity” factor	− 0.067	0.045	2.183	1	0.14	0.936	0.856	1.022
GAF	0.004	0.015	0.055	1	0.815	1.004	0.974	1.034
Overall (score): χ^2^ = 226.838, *df* = 13, *p* = 0.0001								

FEP, first-episode psychosis; AP, antipsychotic; AD, antidepressant; DSM-5, Diagnostic and Statistical Manual for mental disorders, 5th Edition; Baseline acceptance of psychosocial interventions, baseline acceptance of at least individual psychotherapy, family psychoeducation or case management; PANSS, Positive And Negative Syndrome Scale; GAF, Global Assessment of Functioning; *B*, regression coefficient; SE, Standard Error, Wald, Wald statistic value; *df*, degrees of freedom; *p*, statistical significance; HR, Hazard Ratio; 95% CI, 95% Confidence Intervals for HR. Statistically significant p values are in bold

## Discussion

The main aims of this research were to examine short-term disengagement rate in a large Italian population of FEP patients treated within an EIP service across a 12-month follow-up period, and to assess its most significant sociodemographic and clinical predictors at entry.

The results of this investigation showed a 16.5% short-term (1-year) service disengagement rate. This finding is in line with the 15.6% pooled prevalence of disengagement reported in a recent meta-analysis on the strength of engagement in large cohort of 6800 individuals with FEP [5]. However, heterogeneity across studies was very high in this meta-analytic research, with reported disengagement rates ranging from 1% to 41% [63]. Multiple factors and moderators of engagement have been called into question for understanding these prevalence variations [64].

One important reason is the lack of a *consensus definition* of disengagement across investigations [7]. Indeed, it varied broadly from “subjects not in treatment at the end of the research” [65] to “patients terminating treatment despite therapeutic need or untraceable sometimes with a time limit of three months” [66]. It is, therefore, imperative to implement more cohesive methodologies across investigations so that clinical comparisons can be made more accurately. In this respect, according to the evidence-based definition proposed in the meta-analysis by Robson and Greenwood [6], we defined service disengagement as “complete lack of contact or untraceable for three months despite a need for treatment, counted from the date of the last clinical contact”. As also suggested by the authors, we excluded participants who moved out of our catchment area or were “appropriately” discharge (i.e., those FEP participants who “clinically and functionally improved” and were transferred to other private or public, generalist [non-specialized] mental healthcare professionals), as well as FEP people who died or were incarcerated, on the basis that any conclusions about engagement could not be drawn from these events. Among these “early disengagers”, we proposed to dichotomize subjects who actively refused contact with the treatment staff (“active rejecters”) from those who simply did not return phone calls or did not attend appointments despite ongoing therapeutic need (i.e., without an explicit treatment refusal, but silently dropping out the Pr-EP protocol too early and without being no longer traceable) (“faders to black”). This distinction is important because it can identify a subgroup (i.e., faders to black) on which to focus more resources such as home visits, regular contact, and dedicated staff to avoid care interruption.

As to reduce inconsistent findings, other authors [67, 68] suggested to use clinician-rated service engagement scales (such as the “Service Engagement Scale” [SES] or the “Singh-O’Brien Level of Engagement Scale” [SOLES]) [69, 70]. However, the results of the current study showed a significant association between disengagement conceptualized as dichotomous (i.e., presence vs. absence) and a baseline continuous measure of disengagement included in both the HoNOS (i.e., HoNOS “Engagement [historical]” item score) [48] and the Mental Health Clustering Tool (MHCT), which is used in UK clinical practice to measure patient well-being and for the allocation of resources [47].

Another contributor to high sample heterogeneity is the wide variation in *follow-up length*, where shorter studies may capture an artificially inflated disengagement rate including FEP patients who have temporarily dropped out [71, 72]. However, our 1-year service disengagement rate is very similar to the pooled prevalence reported in the (above mentioned) meta-analysis [6] having a 15-month median time to disengage across studies. A 2-year follow-up period will be considered for future papers on the strength of engagement within the Pr-EP program. Finally, other crucial factors contributing to heterogeneity across investigations are related to cultural differences (e.g., belonging to racial-ethnic minority groups) and variations in mental healthcare service models (e.g., how different EIP services operate, the kind of interventions they provide, various catchment populations they served) [7].

### Predictors of short-term disengagement

The results of this research showed that lower baseline acceptance of *psychosocial interventions* was one of the most robust predictors of short-term service disengagement in FEP patients enrolled in the Pr-EP protocol. Together with our reported lower antipsychotic and/or antidepressant prescription rate at entry (i.e., lower acceptance rate of the prescription provided), this finding supports meta-analytic evidence on *poor treatment adherence* as consistent predictive factor related to leaving EIP services [6]. This may reflect low trust in the care that EIP programs offer by the patient and/or family members, or misconceptions about the clinical/therapeutic models offered by some service providers [73]. In this respect, qualitative studies suggested frictions between the subjective meaning that patient gives to experiences of psychosis and the promotion of specific interventions and treatment adherence form a biomedical perspective (“service mismatch”) [74]. Moreover, treatment adherence needs to be understood within a framework of shared decision making, in which patients, family members and EIP staff should find the balance between “the duty to care” (i.e., remaining engage with patients no matter what decision they make) and “the dignity of risk” (i.e., the right to make choice, to fail and to learn) (“aimless engagement: a lack of shared purpose”) [16, 74]. Finally, a “reactive disengagement” in response to individual circumstances (such as medication side effects or a quick returning to work or school) may also exist. In the last case, engagement with EIP service becomes a second priority that young people would follow through with if it does not impact on their primary priority (i.e., the return to work/school) [74, 75].

Anyway, our investigation showed that poor treatment adherence already appears as a *baseline characteristic* of those FEP patients at higher risk of disengagement from the Pr-EP protocol during the first year of treatment. This was also confirmed by the lower baseline HoNOS “Engagement (historical)” item score, suggesting a possible role of less intensive efforts by community mental healthcare professionals for those patients considered to be highly likely to disengage [6]. Identifying and implementing appropriate strategies to improve care motivation, to reduce disengagement or to re-engage the FEP subgroup with no desire to engage and treatment non-adherence (also through remote technologies, telehealth delivery and text messaging) are thus needed [76, 77]. Among these strategies, it is useful to mention specific interventions aimed at favoring patients’ decision making about their therapy and the use of mental health services (such as the shared drawing up of an individualized therapeutic-rehabilitation plan by the subject, her/his family members and health professionals) [44], and/or targeting cognition and motivation in coordinated specialty care for early psychosis (in which social and cognitive training exercises were provided together and within a prolonged supervision by a motivational coach) [78], Moreover, given the usual young age of FEP patients, providing interventions based on their unmet needs and aspirations are also crucial [79].

Another robust predictor of short-term service disengagement from the Pr-EP program was *living with parents*. This parameter is often used as measure that indirectly implies family support [80]. However, our finding showed that family may not necessarily represent a supportive environment, and at the same time that a family supportively involved with treatment might not indicate a service-user internally driven motivation to engage. Moreover, living with family members could also be an indicator of a tendency towards withdrawal and lower propensity for interpersonal relationships, including therapeutic ones. Therefore, family members and caregivers should, therefore, be directly involved in care planning and shared decision making, and encouraged to play their crucial role in promoting engagement (also by reminding FEP patients about clinical appointments, supporting attendance and providing transportation) [81]. When families present barriers to patient’s participation in treatment by arguing against providers’ recommendations and by interfering in participants’ choices, EIP staff should carefully listen to family members, provide support, offer compelling information about psychosis and help participants form their own opinions and navigate family boundaries [7]. In this respect, family psychoeducation and dialogic practices could be of use to better understand interpersonal dynamics, discuss issues from different perspective and implement practical solutions that make sense to the patient and family members [82, 83].

An additional robust predictor in this investigation was the presence of *brief psychotic disorder* or *schizophreniform disorder* at entry. In this respect, drop-out rates of up to 32% were found in FEP patients with brief psychotic disorder [84], who also often do not engage well with individual psychotherapy for psychosis and, therefore, have unmet clinical needs that are not being adequately addressed by current mental healthcare services [85]. Our findings seem to suggest less intensive efforts in engagement or less willingness to take on those FEP patients with a diagnosis of brief psychotic disorder or schizophreniform disorder [85, 86]. Differently, having a diagnosis of schizophrenia at baseline seems to be protective against short-term service disengagement from the Pr-EP program. Thus, greater efforts in defining the diagnosis during the first year of treatment could reduce treatment drop-out and improve care motivation for both FEP patients and family members.

In line with the results reported in the existing literature [6, 8], our investigation supports the role of *migrant status* as predictor of service disengagement. In this respect, it was found that minority groups are less likely to accept a medical model of mental illness, therefore, putting less belief in treatments [87]. Moreover, Anderson and co-workers [80] speculated that ethnic groups may experience increased stigma from their communities and a propensity to deny a need for treatment to fit in with their subjective or cultural norms. In this case, implementing cultural mediation services and cross-cultural oriented interventions to support the engagement of migrants is crucial, also in EIP protocols [88].

The importance of *previous suicide attempt* as additional predictor of short-term service disengagement from the Pr-EP program is not easily interpretable. However, if considered together with the statistical trends observed for lower baseline symptom severity (especially in disorganization, negative symptoms and resistance/excitement-activity dimensions) and for higher daily functioning at entry, this previous feature in the history of FEP patients could be related to a current subjective perception of less seriousness of the clinical picture, a reduced need for treatment, or an actual conviction that attendance takes a lower priority than work, education or leisure activities [6]. As previously suggested, with recent advances in digital technologies, incorporating models of remote or blended delivery could promote engagement on a more casual and convenient basis for FEP individuals, preventing complete discharge.

Finally, in our investigation, marginal predictors of short-term service disengagement (*p* < 0.01) were female gender, low baseline PANSS “Negative Symptoms”, “Disorganization” and “Resistance/Excitement-Activity” factor subscores, as well as high baseline GAF score. Although in need of confirmation, these findings suggest to pay special attention at baseline to female FEP patients, with higher level of functioning and greater clinical severity (especially in terms of excitement, negative and disorganized features).

### Limitations

Several limitations of this investigation should be also acknowledged. First, the lack of an international consensus on disengagement definition limits comparisons across studies and does not allow us to reach generalizable conclusions. Although we used a coherent definition of engagement (as proposed in a recent meta-analysis on service disengagement in FEP populations) [6], it is imperative to implement more cohesive methodologies across investigations. In this respect, Mascayano and colleagues [8] suggested bringing key stakeholders together (e.g., through partnerships) to reach a consensus, to develop common measures of treatment disengagement and to design strategies for increasing engagement when deemed reasonable. This discussion should include providers, FEP individuals and family members, considering that they may have different opinions and perspective.

Second, our research did not account for “true non-engagers” (i.e., those FEP patients who refused any contact with the Pr-EP program or more generally with our generalist mental healthcare services from the start) [7]. Therefore, in the current investigation, participants represented a sub-population of help-seeking people with FEP. This may bias the prevalence of service disengagement if we want to extrapolate the study results to the real-world population with FEP seen in community mental health services. Indeed, it is quite likely that FEP individuals who accepted to be enrolled in the Pr-EP protocol may be more collaborative and engaging than those refusing specialized EIP interventions, so impacting our findings. However, as all FEP help-seekers who were recruited in the Pr-EP program accepted to be included in this research, our results are highly representative of short-term service disengagement rate within a specialized EIP service.

Similarly, the age range (12–35 years), the exclusion of FEP with a DUP of > 2 years, or those FEP patients with previous exposure to antipsychotic drug (such as quetiapine 25 mg for insomnia) may also affect the generalizability of our results.

Finally, our investigation was limited to a 1-year follow-up period. Therefore, our results are comparable exclusively with studies having longitudinally similar designs. Future research with longer follow-up duration to replicate our findings in the Pr-EP program is thus needed.

## Conclusions

The main novelty of this research includes the large sample size and the prospective design. Indeed, several studies reviewed in meta-analyses on service disengagement [6, 7] were conducted on retrospective cohorts. Among prospective cohorts, only few investigations considered large FEP populations with 500 or more participants [3, 22, 23]. In this sense, our findings are robust and represent a good addition to the knowledge on short-term service disengagement.

The results of this research showed that 16% of FEP people enrolled in the Pr-EP program dropped out during the first year of treatment. Particularly robust predictors of short-term service disengagement were baseline treatment non-adherence, living with parents and diagnosis of DSM-5 longitudinally unstable psychotic categories (i.e., brief psychotic disorder or schizophreniform disorder) at entry. There is also evidence that FEP people with migrant status and previous suicide attempt were more vulnerable to disengagement. For these “early disengagers”, a solution might be to remain on EIP program caseloads allowing the option for low-intensity support and monitoring, perhaps via remote technology.

## Data Availability

The data that support the findings of this investigation are available on reasonable request from the corresponding author. The data are not publicly available due to privacy/ethical restrictions.
